# Peripheral neuropathy in limbic encephalitis with anti‐glutamate receptor antibodies: Case report and systematic literature review

**DOI:** 10.1002/brb3.779

**Published:** 2017-08-01

**Authors:** Yi‐Chia Wei, Chin‐Chang Huang, Chi‐Hung Liu, Hung‐Chou Kuo, Jainn‐Jim Lin

**Affiliations:** ^1^ Department of Neurology Chang Gung Memorial Hospital College of Medicine Chang Gung University Keelung Taiwan; ^2^ Community Medicine Research Center Keelung Chang Gung Memorial Hospital Keelung Taiwan; ^3^ Institute of Neuroscience National Yang‐Ming University Taipei Taiwan; ^4^ Department of Neurology Linkou Medical Center Chang Gung Memorial Hospital College of Medicine Chang Gung University Taoyuan Taiwan; ^5^ Graduate Institute of Clinical Medical Sciences Division of Medical Education College of Medicine Chang Gung University Taoyuan Taiwan; ^6^ Division of Pediatric Neurology College of Medicine Chang Gung University Taoyuan Taiwan; ^7^ Chang Gung Children's Hospital Study Group for Children with Encephalitis/Encephalopathy Related Status Epilepticus and Epilepsy (CHEESE) Taoyuan Taiwan

**Keywords:** anti‐AMPA receptor encephalitis, anti‐NMDA receptor encephalitis, glutamate receptor encephalitis, peripheral nervous system, polyneuropathy

## Abstract

**Introduction:**

Autoantibodies to the alpha‐amino‐3‐hydroxy‐5‐methyl‐4‐isoxazolepropionic acid (AMPA) receptor and *N*‐methyl‐d‐aspartate (NMDA) receptor are known to be the causes of autoimmune encephalitis particularly limbic encephalitis. The involvement of the peripheral nervous system is rarely reported.

**Methods:**

We analyzed the serial nerve conduction studies of a previously reported case of anti‐AMPA receptor encephalitis, who was presented with conscious disturbance and quadriplegia. Initial nerve conduction studies (NCS) revealed motor axonal polyneuropathy with active denervation. We also performed systematic review of similar cases with overlapped peripheral neuropathy and glutamate receptor encephalitis through Embase, PubMed, and MEDLINE.

**Results:**

Follow‐up NCS of the patient with anti‐AMPA receptor encephalitis found reverse of the acute neuropathy, which was compatible with clinical recovery of quadriplegia. The systematic review identified 10 cases with overlapping peripheral neuropathy with anti‐AMPA or NMDA receptor encephalitis. Motor or sensorimotor neuropathies were more common than pure sensory neuropathies. Anti‐Hu, anti‐amphiphysin, or anti‐gnaglioside antibodies coexisted in some cases and might be associated with the peripheral symptoms.

**Conclusions:**

Both anti‐AMPA and anti‐NMDA receptor encephalitis could overlap with acute peripheral neuropathy. It is important to consider peripheral symptoms and perform diagnostic tests.

## 1 INTRODUCTION

Alpha‐amino‐3‐hydroxy‐5‐methyl‐4‐isoxazolepropionic acid (AMPA) and *N*‐methyl‐d‐aspartate (NMDA) receptors are two major glutamate receptors and are antigens of patients with autoimmune limbic encephalitis (Lai et al., 2009; Dalmau et al., 2008). B‐cell immunity and antibody‐mediated neuronal dysfunction are pathogenic of these glutamate receptor encephalitis (Peng et al., 2014; Dalmau, Lancaster, Martinez‐Hernandez, Rosenfeld, & Balice‐Gordon, 2011), while role of T‐cell immunity seem to be minor (Liba et al., 2016). Although NMDA and AMPA receptors are two major excitatory synaptic proteins in the central nervous system (CNS), these glutamate receptors also distribute peripherally (Coggeshall & Carlton, 1998). Theoretically, the pathogenic circulating antibodies are able to affect not only the CNS but also the peripheral nervous system (PNS). However, the involvement of the PNS has rarely been emphasized. Our study described serial nerve conduction studies (NCS) of a previously reported patient with anti‐AMPA receptor encephalitis (Wei et al., 2013) and systematically reviewed the peripheral neuropathy in glutamate receptor encephalitis.

## 2 MATERIALS AND METHODS

### 2.1 Clinical presentations and initial NCS of anti‐AMPA receptor encephalitis

In 2012, a 30‐year‐old pregnant woman developed headache (day 1), memory impairment, fever and confusion (day 7), unsteady gait (day 9), quadriparesis (day 11), and myoclonic seizure (day 13). The study of cerebrospinal fluid (CSF) showed that eosinophilic pleocytosis with elevated protein and IgG index (protein 112 mg/dl; white blood cell 70 cells/μl; IgG index 7.38). She was initially treated with antimicrobial agents (acyclovir between day 8 and 19, and albendazole between day 10 and 13) and intravenous dexamethasone (between day 8 and 18). She developed status epilepticus (day 13). She received intravenous anesthesia for status epilepticus (midazolam infusion from day 13 to 15 and propofol infusion from day 13 to 14), and antiepileptic drugs (phenytoin between day 13 and 16, levetiracetam between day 13 and 65, lamotrigine between day 14 and 19, and topiramate between day 19 and 56). Seizure was well controlled without recurrence.

Immunoactivity assay using human embryonic kidney‐293 cells, which were transfected with neuronal surface antigen‐containing plasmids, revealed antibodies to GluA2 subunit of AMPA receptor in both serum and CSF (Wei et al., 2013). She received plasmapheresis (between day 17 and 26), plasma exchange (between day 38 and 46), and methylprednisolone pulse therapy (between day 35 and 36, 52 and 55). However, the patient became stuporous and had quadriplegia, areflexia, and silent plantar responses since intensive care unit (ICU) admission (day 13). The first NCS on day 35 revealed reduced amplitudes of compound muscle action potential (CMAP) in bilateral median, ulnar, tibial, and deep peroneal nerves (Table 1, column 1 m). There was neither conduction block nor reduced conduction velocity of motor nerve conduction. Sensory nerve conduction was intact. The patient was likely to have a motor‐predominant polyneuropathy.

**Table 1:** Serial nerve conduction studies and electromyography

Time from onset		Right			Left			Ref.	Right			Left			Ref.	Right			Left			Ref.
		1 m	2 m	2y	1 m	2 m	2y		1 m	2 m	2y	1 m	2 m	2y		1 m	2 m	2y	1 m	2 m	2y	
**Sensory nerve conduction study**		**Onset latency (ms)**							**Amplitude (mV)**							**Conduction velocity (m/s)**						
Median			1.7		1.0		1.8	***<3.5***		32.3		34		25.7	***>13***		71		50		65	***>50***
Ulnar		1.8	1.6		1.6		1.6	***<3.1***	50	35.7		41		55.5	***>12***	60	63		73		63	***>50***
Sural			2.5	3.1		2.3	2.9	***<3.6***		17.0	9.6		16.6	19.8	***>9***		56	45		61	48	***>38***
**Motor nerve conduction study**		**Onset latency (ms)**							**Amplitude (mV)**							**Conduction velocity (m/s)**						
Median	Wrist	2.9	2.7		2.9	2.8	2.7	***<4.2***	**2.8**	**4.3**		**3.0**	**3.7**	10.7	***>6***							
	Elbow	6.7	6.5		6.6		6.3		**2.8**	**3.1**		**2.8**		10.6		57.9	55		59.5		61	***>50***
Ulnar	Wrist	2.4	2.1		2.3	1.8	2.0	***<3.4***	**2.0**	6.0		**3.3**	**5.3**	13.4	***>5.5***							
	Below elbow	5.8	5.2		5.2		5.2		**0.99**	6.1		**2.3**		13.4								
	Above elbow	7.6	6.7		6.6		7.0		**0.68**	5.9		**2.0**		12.8		55.6	67		71.4		56	***>50***
Deep peroneal	Ankle	4.2	4.1	4.3	3.9	3.1	3.9	***<5.5***	**1.5**	**0.8**	**0.4**	**0.98**	**0.5**	**1.6**	***>2***							
	Below fibular‐head	10.8	10.7	11.5	10.2	9.9	19.3		**0.78**	**0.6**	**0.5**	**0.89**	**0.4**	**0.8**								
	Above fibular‐head	12.6	13.0	13.6	12.2	12.4	22.1		**0.25**	**0.2**	**0.5**	**0.7**	**0.6**	**0.6**		44.4	43	40	40	40	**36**	***>40***
Tibial	Ankle	2.9	3.6	3.9	3.3	3.5	3.2	***<7***	11.3	9.1	15.9	7.0	4.6	15.1	***>4***							
	Popliteal fossa	9.8	11.4	12.0	10.2	11.2	11.1		9.7	7.5	10.8	6.7	4.5	9.4		50.7	49	47	47	49	49	***>40***
**Electromyography** a		**Fibrillations**							**Positive sharp waves**							**Recruitment**						
First dorsal interosseous m			**+++**							**+++**							↓					
Biceps m										**+**							↓					
Anterior tibialis m			**++**							**++**							↓					
Vastus medialis m																	↓					
**Late responses**		**Latency (ms)**																				
Median F‐wave			23.4		**31.5**	25.4	24.8	***<29***														
Ulnar F‐wave		23.1			23.4		22.3	***<30***														
Deep peroneal F‐wave			**NR**	**NR**		**NR**	**NR**	***<50***														
Tibial F‐wave			45.5	45.7		43.8	45.0	***<51***														
H‐reflex			**NR**	**NR**		**NR**	29.7	***<31***														

Ref., referential normal value; m, month; y, year; NR, no response. The numbers listed in bold were abnormal data. The gray numbers in italic were normal values for reference.

The nerve conduction studies (NCS) revealed diffusely decreased amplitudes of motor nerve conductions and poor responses of F‐wave and H‐reflex in the first 2 months (column 1 m and 2 m). There is normal sensory conduction. The electromyography performed at the second month showed fibrillations and positive sharp waves with severely reduced recruitment and normal motor unit potentials of tested muscles, which suggested an active denervation of motor neuropathy (column 2 m). In the first month, the patient was unconscious, quadriplegic with generalized areflexia. At the second‐year follow‐up, she was alert and oriented. Muscle power of all limbs returned to full. The NCS 2 years after onset revealed only peroneal neuropathy (column 2y).

a The motor unit potentials of electromyography were normal.

### 2.2 Systematic literature reviews of peripheral neuropathy in glutamate receptor encephalitis

**Figure 1:**
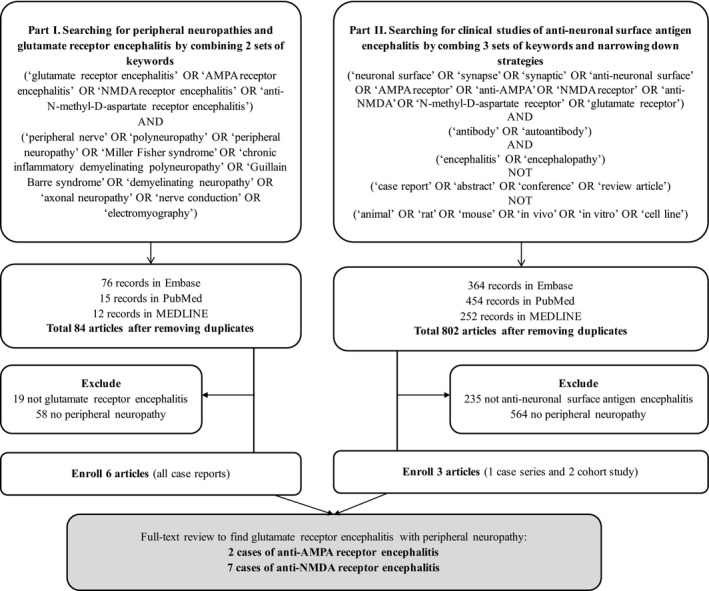
Flowchart of systematic reviews: peripheral neuropathies in patients with glutamate receptor encephalitis. AMPA, alpha‐amino‐3‐hydroxy‐5‐methyl‐4‐isoxazolepropionic acid; NMDA,* N*‐methyl‐d‐aspartate

We conducted systematic reviews of three main databases of medical literatures in English: Embase^**®**^, PubMed^**®**^, and MEDLINE^**®**^. Two searching strategies were applied. First, we searched literatures by combining keywords of glutamate receptor encephalitis and of peripheral neuropathies: (“glutamate receptor encephalitis” OR “AMPA receptor encephalitis” OR “NMDA receptor encephalitis” OR “anti‐N‐methyl‐D‐aspartate receptor encephalitis”) AND (“peripheral nerve” OR “polyneuropathy” OR “peripheral neuropathy” OR “Miller Fisher syndrome” OR “chronic inflammatory demyelinating polyneuropathy” OR “Guillain Barre syndrome” OR “demyelinating neuropathy” OR “axonal neuropathy” OR “nerve conduction” OR “electromyography”). Because narrations of patients' symptoms and signs were commonly covered in contents of case series or cohort studies, the second part of searching used combination of three sets of keywords to find out clinical studies of antineuronal surface antigen encephalitis: (“neuronal surface” OR “synapse” OR “synaptic” OR “anti‐neuronal surface” OR “AMPA receptor” OR “anti‐AMPA” OR “NMDA receptor” OR “anti‐NMDA” OR “N‐methyl‐D‐aspartate receptor” OR “glutamate receptor”) AND (“antibody” OR “autoantibody”) AND (“encephalitis” OR “encephalopathy”). Then, we further narrowed the results down by Boolean logic: NOT (“case report” OR “abstract” OR “conference” OR “review article”) NOT (“animal” OR “rat” OR “mouse” OR “in vivo” OR “in vitro” OR “cell line”). The results from Embase, PubMed, and MEDLINE were merged to remove duplicates and underwent full‐text review. The cases with coexisting peripheral neuropathy were collected (Figure 1).

This study had been approved by the Institutional Review Board of Chang Gung Memorial Hospital and underwent with patient's consensus.

## 3 RESULTS

### 3.1 Serial NCS and clinical follow‐up of the case

The patient received aggressive immunotherapy after diagnosis of anti‐AMPA receptor encephalitis. Her consciousness gradually improved, but quadriparesis and hyporeflexia remained. Therefore, the second NCS and electromyography (EMG) were arranged 2 months after symptom onset (Table 1, column 2 m). Motor amplitudes were reduced, and peroneal F waves and H‐reflex were absent. There is neither conduction block, prolonged latency, nor abnormal sensory conduction. EMG study showed various degree of fibrillations and positive sharp waves with severely reduced recruitment, and normal motor unit potentials in bilateral dorsal interosseous, biceps, and anterior tibialis muscles, suggesting active denervation of diffuse motor axonal neuropathy. She kept receiving immunotherapy and rehabilitation. Her muscle strength returned to normal 1 year after onset. Neuropsychological assessments also revealed significant improvement but remaining mild cognitive impairment (Mini‐Mental State Examination 26/30; impaired in recall 0/3 and orientation 9/10). She only took low‐dose prednisolone 5 mg, bisoprolol 2.5 mg, and amantadine 200 mg per day. The NCS performed 2 years after onset was much improved with only residual peroneal neuropathy (Table 1, column 2y).

### 3.2 Differential diagnoses of the peripheral neuropathy in this case

#### 3.2.1 Myelopathy

Cervical magnetic resonance imaging study was unremarkable.

#### 3.2.2 Metabolic, nutritional, inflammatory, and drug‐induced neuropathies

The laboratory studies did not find diabetes mellitus, renal function impairment (creatinine 0.31 mg/dl; reference 0.44–1.03 mg/dl), abnormal thyroid function (free‐T4 0.96 ng/dl; reference 0.76–1.64 ng/dl), vitamin B12 deficiency (223.2 pg/ml; reference 211–946 pg/ml), porphyria (porphobilinogen 1.44 mg/day; reference 0–2 mg/day), paraproteinemia (negative result in serum protein electrophoresis and immunofixation electrophoresis), vasculitis (negative for antineutrophil cytoplasmic antibody), hepatitis C virus, human immunodeficiency virus infection, syphilis, or heavy metal intoxication by serum tests of lead <0.6 μg/L (reference <23 μg/L), cadmium 1.5 μg/L (reference <2.6 μg/L), mercury <0.9 μg/L (reference <10 μg/L), and arsenic 19.35 μg/g (reference <100 μg/g). She did not have alcohol consumption habit, previous history of polyneuropathy, or hereditary neuropathy in her family. There was also no exposure history to offending agents of drug‐induced neuropathies. Her disease course was similar to that of acute motor axonal neuropathy (AMAN). Although anti‐ganglioside antibodies were not checked in this patient, she did not have common anticipating events of AMAN, such as diarrhea or upper respiratory tract infection. Therefore, she was not likely to have metabolic, nutritional, inflammatory, or drug‐induced neuropathy.

#### 3.2.3 Paraneoplastic syndrome

The surveillance for malignancy included gynecological sonography, breast sonography, pelvis MRI, CSF cytology, peripheral blood smear, tumor markers (CA199 3.12 U/ml, CA153 12.8 U/ml, CEA <0.50 ng/ml, AFP 11.9 ng/ml, SCC 1.70 ng/ml, CA125 487.7 U/ml possibly related to endometriosis, beta HCG 144,559 mIU/ml during pregnancy), and contrast‐enhanced chest computed tomography (after termination of pregnancy). However, no malignant tumor was found.

#### 3.2.4 Critical illness neuropathy and critical illness myopathy

Critical illness neuropathy (CIN) and critical illness myopathy (CIM) are difficult to be distinguished from other acute neuropathies by NCS and EMG studies. Clinical history and laboratory exclusion of other causes are essential. Several predisposing factors are highly correlated to CIN and CIM, including sepsis, multiple organ failure, acute respiratory distress syndrome, ICU admission, and prolonged neuromuscular blocking or sedative agents (Dyck & Thomas, 2005; Katirji, 2002; Hermans, De Jonghe, Bruyninckx, & Van den Berghe, 2008). Although the patient had ICU admission and short‐term midazolam and propofol infusion, gait disturbance and quadriparesis developed before seizure and ICU admission. The absence of sepsis and multiple organ failure suggested low risk of CIN. Most of CINs are axonal type with sensorimotor (60%–71%), followed by pure motor (19%–40%) and pure sensory (0%–10%) pattern (Khan, Harrison, Rich, & Moss, 2006; Zifko, Zipko, & Bolton, 1998). According to the serial NCS, the patient's pure motor neuropathy was the less common type of CIN. Serum creatine kinase (CK) of this patient was normal (160 U/L; reference 20–180 U/L; day 13). Although CIM is usually non‐necrotizing myopathy with limited CK elevation (Hermans et al., 2008), the normal motor unit potentials and severely reduced recruitment of EMG suggested neuropathy rather than myopathy of our patient (Table 1, column 2 m). Therefore, CIN and CIM were less likely to be the cause of the patient's weakness.

### 3.3 Results of systematic review of glutamate receptor encephalitis and peripheral neuropathy

Through the protocol of systematic review (Figure 1), part I searching yielded 76 records in Embase, 15 in PubMed, and 12 in MEDLINE. Full‐text review of 84 merged articles found six case reports of peripheral neuropathy in anti‐NMDA receptor encephalitis (Pohley et al., 2015; Hatano et al., 2011; Tojo et al., 2011; Ishikawa et al., 2013; Pruss, Hoffmann, Stenzel, Saschenbrecker, & Ebinger, 2014; Samejima et al., 2010). One report describing a case with severe axonal neuropathy 33 months before the detection of NMDA receptor antibodies was excluded due to difficulty in identifying the correlation between neuropathy and encephalitis (Köhler et al., 2012). Part II review found 364 records in Embase, 454 in PubMed, and 252 in MEDLINE. One case of anti‐NMDA receptor encephalitis (Byun et al., 2016) and two cases of anti‐AMPA receptor encephalitis were identified (Zekeridou, McKeon, & Lennon, 2016; Hoftberger et al., 2015).

Table 2 summarized the symptoms and NCS/EMG findings of our patient and the nine cases from systematic reviews. Pure motor or motor‐predominant neuropathies were relative common among these cases (4 motor or motor predominant, 3 sensorimotor, 1 pure sensory; Table 2). Response to immunotherapy and reverse of neuropathy were found by serial NCS in at least 2 over 9 cases.

**Table 2:** Systematic literature reviews of cases with overlapped glutamate receptor encephalitis and peripheral neuropathy

Type of antibodies	Reference	Age/sex	CNS symptoms	PNS symptoms	Type of neuropathy by NCS/EMG	Existing of additional antibodies	Tested anti‐neuronal and paraneoplastic antibodies	Tumor	Immunotherapy	Response to treatment
Anti‐AMPA receptor	Hoftberger et al. (2015)	72/M	Short‐term memory loss, ataxia, insomnia, psychotic features	Sensory polyneuropathy	S+/M−, D?/A?	Amphiphysin	NMDAR, AMPAR, GABAbR, LGI1, CASPR2, Hu, Yo, Ri, Ma1/2, Tr, amphiphysin, SOX1, ZIC4, GAD65, AK5, Homer3	Lung cancer	Steroid	Poor, died of cancer
	Zekeridou et al. (2016)	NA	Encephalopathy with limbic or cortical manifestations, insomnia	Peripheral neuropathy, rigidity	NA		NMDAR, AMPAR, GABAbR, GABAaR, VGKC, CASPR2, mGluR1, mGluR5, GlyR, AQP4, AChR	Thymoma	NA	NA
	Wei et al. (2013) (This case)	30/F	Short‐term memory loss, ataxia, confusion, coma	Quadriplegia, areflexia	S−/M+, D‐/A+ Reversible		NMDAR, AMPAR, GABAbR, LGI1, CASPR2, GAD65	None	Steroid/PE/PP/Azathioprine	Good
Anti‐NMDA receptor	Samejima et al. (2010)	75/M	Memory loss, ophthalmoplegia, seizure	Distal weakness and muscle atrophy of limbs	S+/M+ motor predominant, D+/A+	Hu	NMDAR, Hu, Yo, Ri, Ma, CV2, amphiphysin	Lung cancer	IVIg	Poor, died of cancer
	Hatano et al. (2011)	23/F	Ophthalmoplegia, nystagmus, ataxia, orolingual dyskinesia, psychosis	Ataxia, areflexia, atypical Miller–Fisher syndrome.	NA	GQ1b, GT1a	NMDAR, GQ1b, GT1a (complete panel not listed)	None	Steroid/IVIg	Good
	Tojo et al. (2011)	19/M	Psychomotor agitation, orolingual dyskinesia, status epilepticus	Preceding Guillain‐Barré Syndrome	S−/M+, D+/A−		NMDAR, GM1, GQ1b	None	Steroid/IVIg	Partial
	Ishikawa et al. (2013)	26/F	Ophthalmoplegia, confusion, hypoventilation, dysautonomia,	Flaccid paraplegia	S−/M+, D?/A? Reversible		NMDAR, VGKC, GM1, GD1a, GD1b, GQ1b, GT1a	Ovarian teratoma	Steroid/IVIg	Good
	Pruss et al. (2014)	75/M	Agitation, hyperkinetic movement, fever, respiratory failure	Lower limb predominant ascending pain and numbness, difficulties of walking	S+/M+, D+/A+		NMDAR, AMPAR, GABAbR, LGI1, CASPR2, GlyR, AQP4, Tr, Hu, Yo, Ri, Ma/Ta, amphiphysin, GAD65, MAG, myelin	None	Steroid/IVIg/PE/rituximab	Partial
	Pohley et al. (2015)	32/M	Headache, hallucination, ataxia, ophthalmoplegia, anisocoria, seizure	Lower limb hyperesthesia and areflexia. Myositis with myalgia and muscle atrophy	S+/M+, D+/A−	Hu	NMDAR, Hu (Euroimmun, complete panel not listed)	Primary mediastinal seminoma	Steroid/IVIg/PP	Poor
	Byun et al. (2016)	67/M	Visual hallucination, ataxia	Polyneuropathy pattern	NA	Hu	NMDAR, AMPAR, GABAbR, LGI1, CASPR2, Hu, Yo, Ri, Ma2, CV2/CRMP5, Amphiphysin	NA	NA	NA

AMPA, alpha‐amino‐3‐hydroxy‐5‐methyl‐4‐isoxazolepropionic acid; NMDA, *N*‐methyl‐d‐aspartate; M, male; F, female; NA, not available; NCS, nerve conduction study; EMG, electromyography; S, sensory; M, motor; D, demyelinating; A, axonal; NMDAR, NMDA receptor; AMPAR, AMPA receptor; GABA_B_ R, γ‐aminobutyric acid B receptor; GABA_A_R, γ‐aminobutyric acid A receptor; LGI1, leucine‐rich glioma inactivated protein 1; CASPR2, contactin‐associated protein‐like 2; VGKC, voltage‐gated potassium channel; ZIC, zinc finger protein; GAD65, 65 kDa glutamic acid decarboxylase; mGluR, metabotropic glutamate receptor; GlyR, glycine receptor; AQP4, aquaporin‐4; AchR, acetylcholine receptor; MAG, myelin‐associated glycoprotein; IVIg, intravenous immunoglobulin; PE, plasma exchange; PP, plasmapheresis.

A total of three cases of anti‐AMPA receptor encephalitis (2 from literature review and 1 from our case) and seven cases of anti‐NMDA receptor encephalitis were identified.

## 4 DISCUSSIONS

The serial NCS/EMG findings of our patient suggested that acute reversible motor axonal polyneuropathy could coexist with acute anti‐AMPA receptor encephalitis. We also found nine similar cases combining acute neuropathies with acute anti‐AMPA or NMDA receptor encephalitis in systematic literature review. Anti‐NMDA receptor encephalitis may also overlap with other diseases. Demyelinating diseases including neuromyelitis optica spectrum disorder (NMOSD) (Titulaer et al., 2014), brain stem encephalitis, leukoencephalopathy following herpes simplex encephalitis, and acquired demyelination syndrome could overlap with anti‐NMDA receptor encephalitis (Hacohen et al., 2014). Coexisting antibodies including antibodies against aquaporin‐4 (AQP4) and myelin oligodendrocyte glycoprotein (MOG) were found in the patients who had overlapping anti‐NMDA receptor encephalitis and NMOSD (Titulaer et al., 2014). From our systematic literature review, anti‐Hu antibody was found to be the coexisting antibody in three cases and was assumed to be the paraneoplastic antibody in two of them (Pohley et al., 2015; Samejima et al., 2010). The clinical manifestations of these cases were typical for anti‐Hu antibody associated neuropathy (Camdessanche et al., 2002). Besides, antibodies against amphiphysin was noted in another case with anti‐AMPA receptor encephalitis and was assumed to be the paraneoplastic antibodies of lung cancer (Hoftberger et al., 2015; Saiz et al., 1999). Antibodies to ganglioside were also found in another reported case with anti‐NMDA receptor encephalitis (Hatano et al., 2011). However, this case probably had overlapped Miller–Fisher syndrome (Kaida et al., 2006). In summary, the presence of coexisting antibodies may indicate an overlapping syndrome. It is important to arrange diagnostic tests like NCS/EMG in anti‐AMPA and anti‐NMDA receptor encephalitis patients when peripheral nervous system involvement is suspected. It is also important to arrange comprehensive work‐ups of possible malignancy when these coexisting antibodies are well‐known paraneoplastic antibodies.

Another reasonable anatomical explanation of our patient's presentation might be motor neuron dysfunction due to delayed late response with pure motor involvement. Both NMDA receptors (Spalloni, Nutini, & Longone, 2013) and AMPA receptors (Tomiyama et al., 1996) are not only widespread in the neocortex, but also are distributed in anterior horn cells of the spinal cord. Antibody‐mediated damage on motor neurons had been found in autopsy of a case with anti‐NMDA receptor encephalitis (Tuzun et al., 2009). AMPA receptor GluA2 subunit RNA editing error also played roles in developing motor neuron disease (Hideyama et al., 2012). Therefore, motor neuron injury in acute anti‐AMPA receptor encephalitis could be another possible cause of motor paresis. Immunohistological statins or animal studies are warranted in the future for further confirmation.

To date, concurrent motor paresis or peripheral presentations had rarely been described in anti‐AMPA receptor encephalitis. (Lai et al., 2009) only reported one patient with rigidity due to coexisting stiff‐person syndrome and another with gait disturbance in a case series of 10 patients. Besides, anticipating Guillain–Barré syndrome (GBS) had also been reported in patients with anti‐NMDA receptor encephalitis (Tojo et al., 2011; Pruss et al., 2014). Moreover, GBS may also develop concurrently with acute disseminated encephalomyelitis (Bernard, Riou, Rosenblatt, Dilenge, & Poulin, 2008), Bickerstaff's brainstem encephalitis (Odaka et al., 2003; Han et al., 2012), and combined central and peripheral demyelination (Ogata et al., 2016; Cortese et al., 2016). In clinical practice, peripheral symptoms may be masked by severe CNS dysfunctions (Joubert et al., 2015). Therefore, localization of motor weakness requires comprehensive history taking, neurological examination, assistance of electrophysiological tools, and neuroimaging. However, differentiating overlapping syndromes from patients with concurrent CNS and PNS disorders is still challenging. We wish our study will help to improve the detection of PNS involvement in glutamate receptor encephalitis in the future.

This literature review was based on previously published reports. Due to the rarity of this disease, the majority of articles were case reports and the clinical correlations were difficultly studied. For example, although patients with underlying malignancies (e.g., two lung cancers and one seminoma in Table 2) had poor outcomes in our review, registry studies with larger patient numbers in the future may be needed to examine the statistical correlation. Second, the literature searching was based on full‐text or abstract in English and publications in other languages could also be missed. Third, some paraneoplastic autoantibodies including anti‐Hu antibody were not examined in this patient, which may lead to bias of our results.

## 5 CONCLUSIONS

PNS and CNS presentations could overlap in anti‐AMPA receptor and anti‐NMDA receptor encephalitis. It is important to arrange proper diagnostic tests when peripheral neuropathy is considered.

## CONFLICT OF INTEREST

All authors declare that there's no conflict of interest existed.
